# Regression of Concurrence via Local Unitary Invariants

**DOI:** 10.3390/e26110917

**Published:** 2024-10-29

**Authors:** Ming Li, Wenjun Wang, Xiaoyu Zhang, Jing Wang, Lei Li, Shuqian Shen

**Affiliations:** College of Science, China University of Petroleum, Qingdao 266580, China; liming3737@163.com (M.L.); wwjun0920@163.com (W.W.); tazxy@126.com (X.Z.); lileiupc@126.com (L.L.); shuqianshen@126.com (S.S.)

**Keywords:** concurrence, Local unitary invariants, regression

## Abstract

Concurrence is a crucial entanglement measure in quantum theory used to describe the degree of entanglement between two or more qubits. Local unitary (LU) invariants can be employed to describe the relevant properties of quantum states. Compared to quantum state tomography, observing LU invariants can save substantial physical resources and reduce errors associated with tomography. In this paper, we use LU invariants as explanatory variables and employ methods such as multiple regression, tree models, and BP neural network models to fit the concurrence of 2-qubit quantum states. For pure states and Werner states, by analyzing the correlation between data, a functional formula for concurrence in terms of LU invariants is obtained. Additionally, for any two-qubit quantum states, the prediction accuracy for concurrence reaches 98.5%.

## 1. Introduction

The concept of quantum entanglement was first introduced as a peculiar physical phenomenon in the early stages of quantum mechanics, demonstrating a unique connection between two or more quantum systems [1]. It holds significant importance in the field of quantum cryptography, quantum teleportation, and quantum computing. Because of its importance, detecting and quantifying entanglement have been pivotal tasks in quantum information processing [2,3,4,5,6]. In [7,8], the authors have proposed experimentally feasible direct detection of quantum entanglement based on the separability criterion of positive maps and the direct measure of a nonlocal wave function of a bipartite system using modular values, respectively.

Currently, scholars have proposed numerous highly practical measures of entanglement for various scenarios, such as entanglement formation [9], negativity [10], and the relative entropy of entanglement [11], tailored to the nature of the research question. Among these entanglement measures, concurrence is a highly important one in quantum theory, which serves as an effective tool in studying quantum entanglement, enabling the description of quantum phase transition in many-body quantum systems’ interactions [12,13]. Furthermore, the value of concurrence can be used to estimate the formation of entanglement [14]. In 1997, Wootters et al. proposed an elegant formula in Ref. [15] for calculating the concurrence of any arbitrary two-qubit quantum state as follows:

(1) $$\begin{array}{c} C\left(\rho \right)=\max\left\{\lambda_{1}-\lambda_{2}-\lambda_{3}-\lambda_{4},0\right\}, \end{array}$$

where $\lambda_{i}$ are the non-negative square roots, in descending order, of the eigenvalues of the non-Hermitian matrix $\rho \tilde{\rho }$. And $\tilde{\rho }=\left(\sigma_{y}⊗\sigma_{y}\right)\rho^{*}\left(\sigma_{y}⊗\sigma_{y}\right)$, where $\rho^{*}$ is the complex conjugate of matrix ρ and $\sigma_{y}$ stands for the Pauli-Y matrix.

However, in order to compute the concurrence of a bipartite system, it is necessary to possess complete information about the system’s density matrix, which can be achieved through quantum tomography. Even though the density matrix structure of a two-qubit quantum system is not excessively intricate, it still demands a certain quantum resource allocation and may entail measurement errors during tomography [16,17,18]. Therefore, we aim to describe quantum systems using local unitary (LU) invariants of quantum states to mitigate errors introduced by tomography. In this scenario, prior knowledge of the system’s density matrix is not required. It suffices to determine the system’s Hamiltonian and subject it to specific transformations to compute these LU invariants.

In this paper, we endeavor to use machine learning techniques, including multivariate regression models, tree models, and neural network models, to uncover the mathematical relationship between LU invariants and concurrence within two-qubit systems. Our objective is to establish a model capable of estimating the degree of entanglement solely based on LU invariants, thus facilitating entanglement characterization without prior knowledge of the density matrix.

## 2. LU Invariants of Two-Qubit States

Before introducing LU invariants, let us first describe quantum states using Bloch representation. The Bloch representation of a two-qubit state can be then expressed as follows:

(2) $$\begin{array}{cc} \rho = & \frac{1}{4}I⊗I+\frac{1}{2}\sum_{i=1}^{3}s_{i}\;\sigma_{i}⊗I\\ \; & +\frac{1}{2}\sum_{j=1}^{3}p_{j}\;I⊗\sigma_{j}+\sum_{i,j=1}^{3}{\hat{\beta }}_{ij}\;\sigma_{i}⊗\sigma_{j}, \end{array}$$

where $\sigma_{i},i=1,2,3$ represent the Pauli-X, Pauli-Y, and Pauli-Z matrices, respectively, and

$$\begin{array}{cc} s= & \frac{1}{2}\left[\mathrm{tr}\left(\rho \sigma_{x}⊗I\right),\mathrm{tr}\left(\rho \sigma_{y}⊗I\right),\mathrm{tr}\left(\rho \sigma_{z}⊗I\right)\right],\\ p= & \frac{1}{2}\left[\mathrm{tr}\left(\rho I⊗\sigma_{x}\right),\mathrm{tr}\left(\rho I⊗\sigma_{y}\right),\mathrm{tr}\left(\rho I⊗\sigma_{z}\right)\right],\\ \hat{\beta }= & \frac{1}{4}[\mathrm{tr}\left(\rho \cdot \sigma_{x}⊗I\cdot I⊗\sigma_{x}\right),\mathrm{tr}\left(\rho \cdot \sigma_{x}⊗I\cdot I⊗\sigma_{y}\right),\\ \; & \;\;\;\mathrm{tr}(\rho \cdot \sigma_{x}⊗I\cdot I⊗\sigma_{z});\\ \; & \;\mathrm{tr}\left(\rho \cdot \sigma_{y}⊗I\cdot I⊗\sigma_{x}\right),\mathrm{tr}\left(\rho \cdot \sigma_{y}⊗I\cdot I⊗\sigma_{y}\right),\\ \; & \;\;\;\mathrm{tr}(\rho \cdot \sigma_{y}⊗I\cdot I⊗\sigma_{z});\\ \; & \;\mathrm{tr}\left(\rho \cdot \sigma_{z}⊗I\cdot I⊗\sigma_{x}\right),\mathrm{tr}\left(\rho \cdot \sigma_{z}⊗I\cdot I⊗\sigma_{y}\right),\\ \; & \;\;\;\mathrm{tr}(\rho \cdot \sigma_{z}⊗I\cdot I⊗\sigma_{z})]. \end{array}$$

In Ref. [19], Makhlin proved that, in general, two two-body quantum states are local unitary equivalent if and only if the following 18 invariants (see Table 1 in detail) are equal.

Due to concurrence also exhibiting local unitary invariance, we seek to describe each 2-qubit state using this set of LU invariants. Concurrence is a significant measure of entanglement for m⊗n(m⩽n)-dimension bipartite systems. For a bipartite pure state $|\varphi \rangle \in H_{AB}=H_{A}⊗H_{B}$, where $H_{A}$ ($H_{B}$) denotes the *m* (*n*)-dimensional vector space associated with the subsystem *A* (*B*) such that m≤n, the concurrence [15,20] is defined by

$$\begin{array}{c} C_{A|B}\left(|\varphi \rangle \right)=\sqrt{2\left(1-\mathrm{tr}\rho_{A}^{2}\right)}, \end{array}$$

with the reduced matrix $\rho_{A}$ obtained by tracing over the subsystem *B*, and tr(·) denotes the tracing of the density matrix. The concurrence is then extended to mixed states $\rho \in H_{AB}$ by the convex roof:

(3) $$C_{A|B}\left(\rho \right)\equiv \min_{\left\{p_{i},|\varphi_{i}\rangle \right\}}\sum_{i}p_{i}C_{A|B}\left(|\varphi_{i}\rangle \right),$$

where the minimum is taken over all possible ensemble decompositions of $\rho =\sum_{i}p_{i}|\varphi_{i}\rangle \langle \varphi_{i}|$, $p_{i}\geq 0$ and $\sum_{i}p_{i}=1$. The value of concurrence ranges from 0 to 2(m−1)/m, where a concurrence of 0 indicates a separable state, values greater than 0 signify the presence of entanglement within the system, and 2(m−1)/m represents a maximally entangled state. For two-qubit states, estimating its concurrence using machine learning methods can be viewed as a regression problem with values ranging from 0 to 1.

## 3. Constructing Regression Models

Machine learning is a method for extracting patterns, trends, and correlations from large-scale noisy data. By analyzing the features and patterns within the data, machine learning algorithms can automatically uncover hidden information and learn from it. This learning process enables machines to continuously improve and optimize their performance to better handle unknown data and scenarios. Faced with vast and complex datasets, machine learning becomes a powerful tool for solving real-world problems and making predictions. In this paper, the models we use are the multiple linear regression model, the BP neural network [21], and the two tree models, eXtreme Gradient Boosting (XGBoost) [22] and Light Gradient Boosting Machine(LGBM) [23]. The metrics of the assessment model adopted in this paper are $R^{2}$ and RMSE, and the definitions are as follows:

$$\begin{array}{c} RMSE=\sqrt{\frac{1}{n}\sum_{i=1}^{n}{\left(y_{i}-{\hat{y}}_{i}\right)}^{2}}, \end{array}$$

$$\begin{array}{c} R^{2}=\frac{SSR}{SST}=\frac{\sum {\left({\hat{y}}_{i}-\bar{y}\right)}^{2}}{\sum {\left(y_{i}-\bar{y}\right)}^{2}} \end{array}$$

where *n* is the sample size, $y_{i}$ is the actual observations, ${\hat{y}}_{i}$ is the model predictions, and $\bar{y}$ is the mean of the observations. The smaller the RMSE, the closer the $R^{2}$ is to 1, the better the model explains the dependent variable.

In the following, we aim to use data analysis methods to input LU invariants as explanatory variables into an algorithmic model and fit the values of concurrence.

Due to the varying structures of density matrices representing quantum states, which reflect different properties and complexities of quantum systems, this experiment will progress from specific to general quantum state structures. Specifically, fitting concurrence will be conducted starting from Werner states, followed by pure states, and then mixed states.

### 3.1. For Werner States

The Werner states are a family of quantum states with a particular structure, proposed by R. Werner in 1989 [24]. It is a type of two-qubit quantum state with highly symmetric properties. The density matrix expression for the two-qubit Werner state $\rho_{w}$ is as follows:

(4) $$\begin{array}{c} \rho_{w}=p|\psi \rangle \langle \psi |+\frac{\left(1-p\right)}{4}I \end{array}$$

where $|\psi \rangle =\frac{1}{\sqrt{2}}(|01\rangle -\left|10\rangle \right)$, representing the Bell state; *I* denotes the four-dimensional identity matrix; and *p* represents a real coefficient with 0⩽p⩽1. The value of *p* describes the evolution of the quantum system from a mixed state to a pure state. The degree of entanglement of the quantum system depends on the value of *p*: when p⩽1/3, the Werner states are separable states; when p>1/3, the Werner states are entanglement states; and when p=1, the Werner states represent the maximal entangled states, i.e., Bell states.

For two-qubit Werner states, we can construct a family of quantum states by setting different values of *p*. Due to its specific structure, we can directly derive its concurrence using LU invariants. In Ref. [25], Chen et al. proposed that the concurrence of Werner states is dependent on their parameters, and its formula is given by the following:

(5) $$\begin{array}{c} C\left(\rho_{w}\right)=\max\left\{-\frac{1}{2}+\frac{3}{2}p,0\right\},\;0⩽p⩽1. \end{array}$$

Accordingly, the LU invariant of the Werner state can be calculated,

$$\begin{array}{c} s=\left(0,0,0\right),p=\left(0,0,0\right),\hat{\beta }=\frac{1}{4}\left(\begin{array}{ccc} -p & 0 & 0\\ 0 & -p & 0\\ 0 & 0 & -p \end{array}\right). \end{array}$$

Therefore, the formula can be obtained by using LU invariants to express the degree of concurrence

(6) $$\begin{array}{c} C\left(\rho_{w}\right)=\max\left\{-\frac{1}{2}+2\sqrt{3I_{2}},\;0\right\}. \end{array}$$

### 3.2. For Pure States

Pure states refer to quantum systems being in definite and precise states, where the pure states’ system will always collapse to specific measurement outcomes with deterministic probabilities under any measurement. Its density matrix can be represented by a state vector |ψ〉 as

$$\begin{array}{c} \rho =|\psi \rangle \langle \psi |. \end{array}$$

According to the definition of pure states, we randomly generate complex vectors in four dimensions where the complex vector consists of a real part and an imaginary part, both of which are randomly drawn from a uniform distribution. Next, dividing it by its L2-norm, we obtain normalized complex vectors |ψ〉. We calculate its concurrence using Equation (1) as the target value for our model. Additionally, we compute 18 LU invariants of the quantum state as explanatory variables. We generate a total of 100,000 quantum states as experimental data.

**Figure 1:**
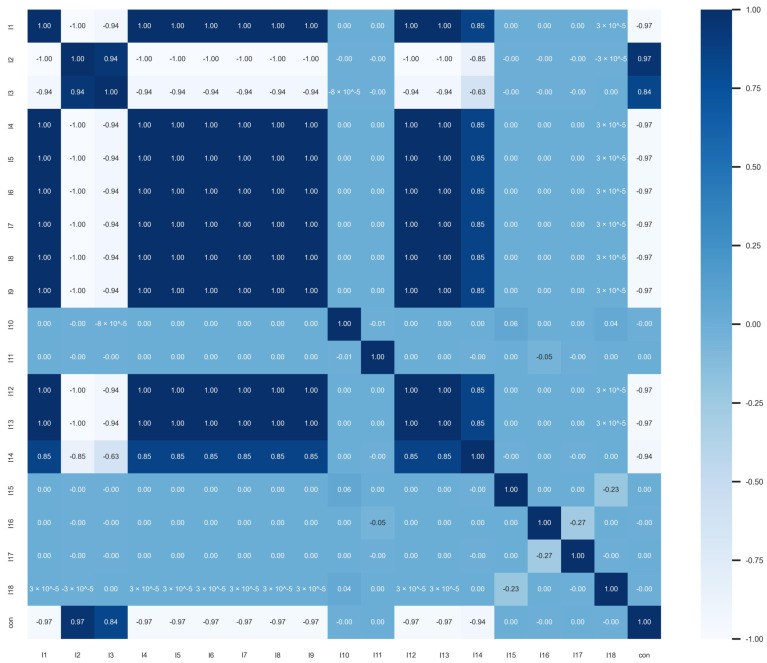
The figure shows a heatmap illustrating the linear correlation between 2−qubit pure state LU invariants and concurrence. In the heatmap, the linear correlation between concurrence and both $I_{1}-I_{9}$ and $I_{12}-I_{14}$ is particularly high.

First, we calculate the correlation coefficient to check if there is a linear correlation between the variables. The results are presented in the form of a heatmap, as shown in Figure 1. For two-qubit pure states, there is a strong linear relationship between LU invariants and concurrence, such as between variables $I_{1}$, $I_{2}$, $I_{4}-I_{9}$, and $I_{12}-I_{14}$. Additionally, based on the calculation results, there is also a strong auto correlation between the explanatory variables. For example, $I_{1}$ and $I_{2}$, and $I_{4}-I_{9}$, and $I_{12}$ and $I_{13}$ exhibit a linear correlation of 1. We selected a penalized multivariate regression model, a nonlinear tree model, and a neural network model for model fitting.

The experimental data are split into training and testing sets in an 8:2 ratio, randomly selecting 80% of the samples for the training set and 20% for the testing set. Next, the models are fitted, and the evaluation results on the testing set are shown in Table 2. All five models demonstrated good performance.

**Figure 2:**
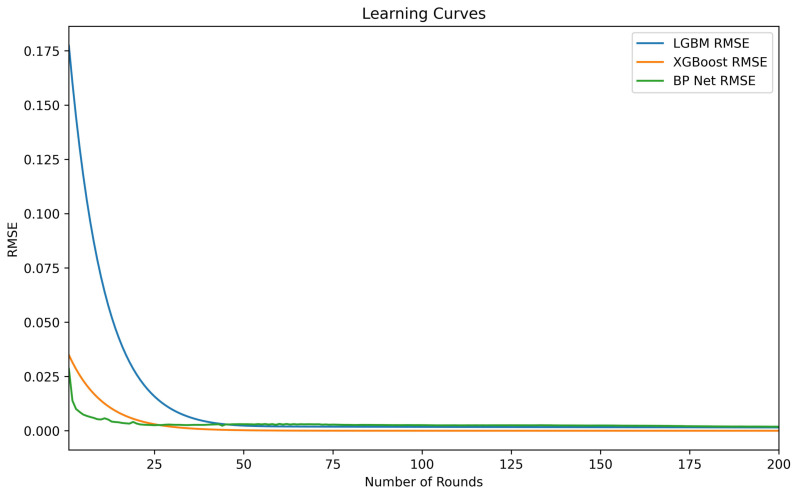
The learning curves for the 2-qubit pure state models show that the BP-Net converges the fastest, followed by XGBoost.

When fitting the model using a linear approach, we employed the penalized Lasso model due to multicollinearity among the variables. Compared to the multivariate linear regression model, the Lasso model reduced the multicollinearity among the independent variables, though at the cost of some accuracy, resulting in an R-squared value of 0.8779. When using nonlinear tree models for fitting, both the XGBoost and LGBM models exhibited better fitting performance than the linear model, with R-squared values reaching 0.9999. As shown in the learning curves of the training models in Figure 2, the XGBoost model converged faster than the LGBM model. Although the BP neural network model has the lowest RMSE of $1.57\times 10^{-5}$, its fitting performance was not as superior as the tree models.

**Figure 3:**
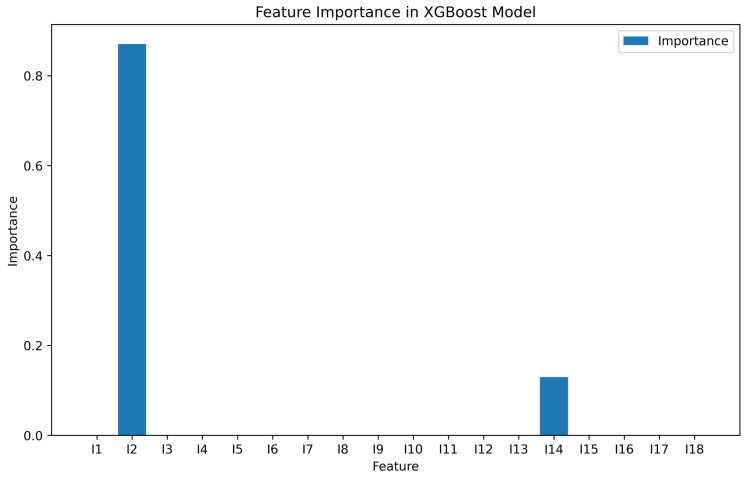
The feature importance values from the XGBoost model indicate that concurrence is related to $I_{2}$ and $I_{14}$, as shown in the figure.

**Figure 4:**
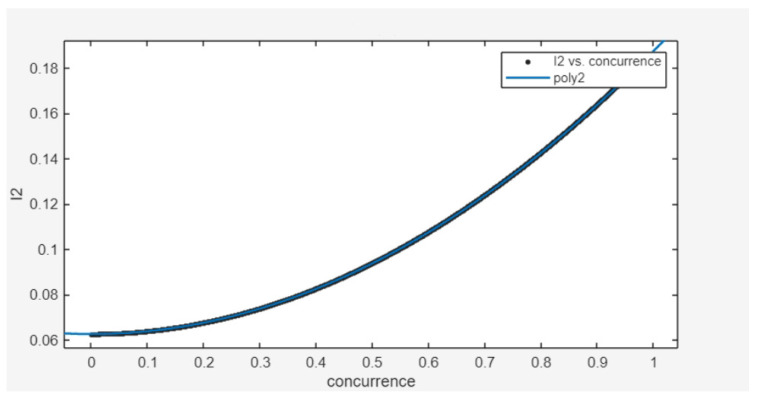
Using Matlab(R2024a)’s Curve Fitting Tool to fit a function between concurrence and the variable $I_{2}$, when $I_{2}$ is the target variable and concurrence is the descriptive variable, it fits a quadratic polynomial. The fitting coefficient is 1, and the RMSE is nearly 0.

Since XGBoost demonstrated superior performance in the previous fitting, we output the feature importance of the model, as shown in Figure 3. In the XGBoost model, the concurrence of a 2-qubit pure state is related to the variables $I_{2}$ and $I_{14}$. Therefore, we hypothesize that there must be some nonlinear relationship between the concurrence of a 2-qubit pure state and the variables $I_{2}$ and $I_{14}$. After trying both primary and binary quadratic polynomials, it was found that the quadratic polynomial, which is satisfied when $I_{2}$ is used as the target variable and concurrence is used as the descriptor variable, was the most superior performing fit, as shown in Figure 4.

The functional relationship is as follows:

$$\begin{array}{c} y=\frac{1}{8}x^{2}+\frac{1}{16} \end{array}$$

Further, the functional relationship between $I_{2}$ and concurrence can be obtained:

$$\begin{array}{c} C\left(\rho \right)=\max\left\{\sqrt{8I_{2}-\frac{1}{2}},0\right\} \end{array}$$

For this formula, we hope to achieve a more rigorous mathematical derivation in future research. Next, we will fit the concurrence for the general quantum states’ structure.

### 3.3. For General Structure States

To generate general two-qubit quantum states, we express the density matrices as follows:

$$\begin{array}{c} \rho =\frac{M^{†}M}{\mathrm{tr}(M^{†}M)} \end{array}$$

where *M* is a randomly generated complex matrix where the real and imaginary parts of the elements in *M* are randomly drawn from a uniform distribution. Based on the above equation, we randomly generate 100,000 complex density matrices and calculate the 18 LU invariants and the concurrence of each density matrix as the experimental data. A boxplot is then drawn to observe the distribution of the variables.

**Figure 5:**
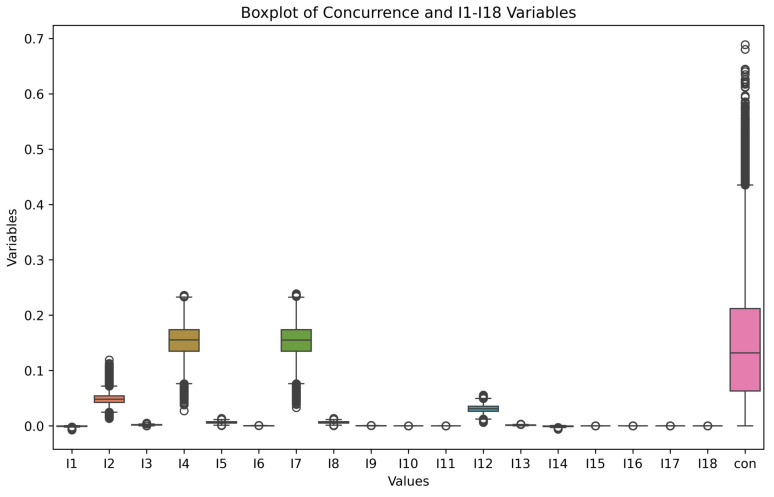
The boxplot shows the numerical distribution of 2-qubit quantum state variables calculated using normalized **s**, **p**, and $\hat{\beta }$.

As shown in Figure 5, the concurrence of generated quantum states is mostly concentrated between 0 and 0.45. This is because the generated quantum states are mostly mixed states, and the entanglement of mixed states is weaker compared to pure states. The distributions of the invariants $I_{3}$, $I_{6}$, $I_{9}$, and $I_{13}$ are more varied; the distributions of $I_{1}$, $I_{5}$, $I_{8}$, $I_{12}$, and $I_{14}$ are less varied; all the values of $I_{2}$, $I_{4}$, and $I_{7}$ are 1 because the 2-norms are themselves; and the values of the invariants $I_{10}$, $I_{11}$, and $I_{15}-I_{18}$ fluctuate around 0.

**Figure 6:**
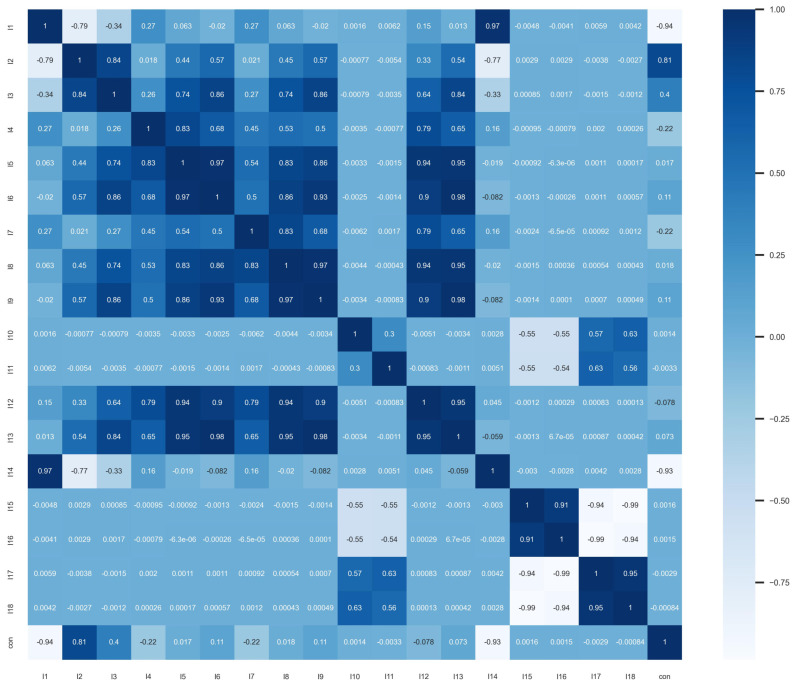
Heatmap of correlation coefficients of 2−qubit quantum state variables; there is no obvious linear relationship between the variables and concurrence.

**Figure 7:**
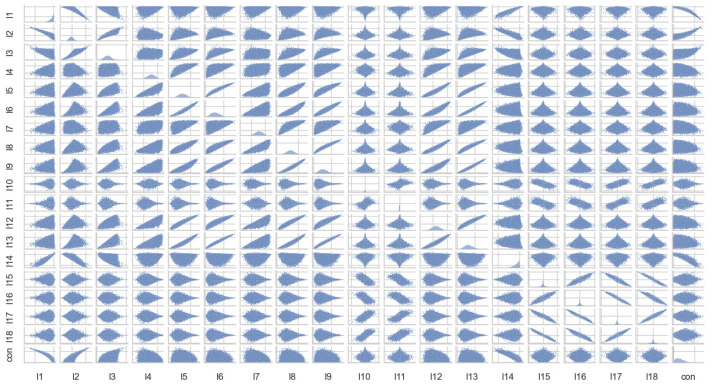
Scatterplot of 2−qubit quantum state variables.

The scatterplot and correlation coefficient are then used to observe the linear correlation between the variables. As shown in Figure 6 and Figure 7, the concurrence of the target variable only has an obvious linear relationship with $I_{1}$, $I_{2}$, and $I_{14}$, and the independent variables all have different degrees of linear correlation.

We substitute data into multiple linear regression models, the Lasso model, XGBoost model, LGBM model, and BP neural network model, for training. The performance results of the models on the test set are shown in Table 3. Due to the complexity of the universal quantum state density matrix structure, compared with the results of the simple pure state and the Werner state with a special structure, the fitting result of the multiple linear regression model is also great. However, due to the possibility of multicollinearity in the model, the model is overfitted. Therefore, the Lasso model is used to reduce the collinearity, and the decision coefficient of the model after fitting is only 0.8302. The tree model with the nonlinear structure XGBoost and LGBM still have the best fitting effect, with decision coefficients of 0.9665 and 0.9717, respectively, followed by the BP neural network model with a decision coefficient of 0.9488.

In the following, we try to optimize the model.

Since there is no obvious linear relationship between the variables, we no longer consider using a linear model to fit the degree of concurrence.

**Figure 8:**
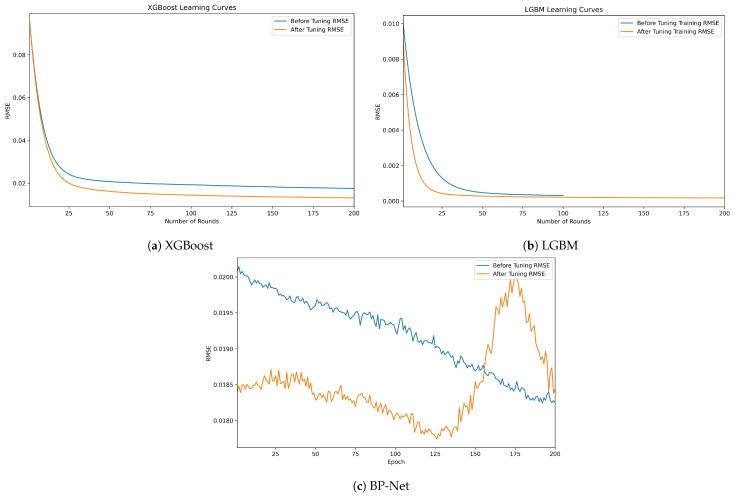
Model learning curve after parameter tuning.

For the tree model, the grid search algorithm is used to find the optimal parameters of the model, that is, to set the candidate value list of the hyperparameters, we traverse all the hyperparameters to combine them, train and evaluate each group of hyperparameters, and finally select the best performing hyperparameter combination as the hyperparameter of the model. The hyperparameters of the XGBoost model and LGBM model are shown in Table 4. The learning curve after parameter adjustment is shown in Figure 8. The blue one is the initial learning curve, and the orange one is the learning curve after parameter adjustment. The RMSE of the XGBoost model decreases significantly, and the convergence speed of the LGBM model also increases significantly.

For the neural network model, grid search is also used to determine the hyperparameters of the model: the ReLU function is selected as the hidden layer activation function, four fully connected layers are set, Adam is used as the model optimizer, and the learning curve becomes stable when the number of iterations is 200 so as to build the final neural network model.

**Figure 9:**
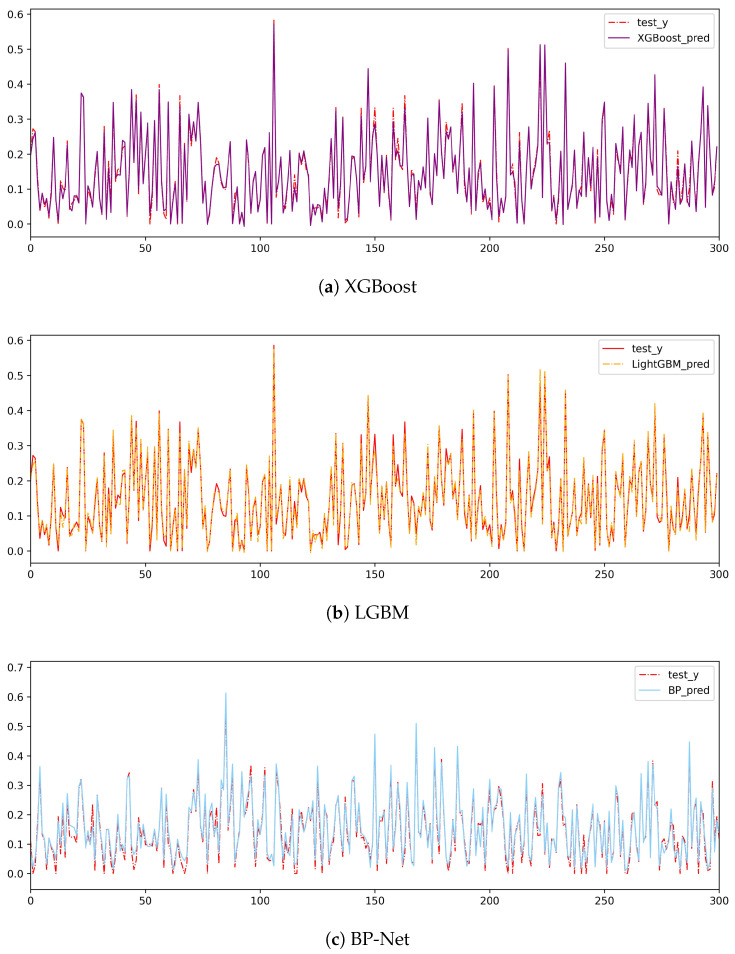
Boxplot of the distribution of 2-qubit quantum state variables.

After the test set is substituted into the model to calculate the score, the results of which are shown in Table 5, the decision coefficients of the optimized model are improved to varying degrees. Among them, the tree model has the best fitting effect, followed by the BP neural network model. The comparison curve between the predicted value of the model and the true value is shown in Figure 9. Then, the pure state and Werner state of the special structure are substituted into the model for fitting. In the generated state, LGBM is the best performer, with the lowest RMSE and $R^{2}$ of 0.9855. XGBoost and BP-Net also perform better but slightly less well than LGBM. In the pure state, XGBoost and LGBM are very close to each other, with a very low RMSE and an R² of nearly 1. In the Werner state, all the models have a very low RMSE and an R² of nearly 1, which indicates that the models perform very well in the Werner state.

## 4. Conclusions and Discussion

Using classical machine learning to solve quantum information problems is one of the hot issues in current research. Classical machine learning methods provide more perspectives for exploring the structure of quantum states and studying the properties of quantum states. In this chapter, LU invariants are used as descriptor variables to train the model and predict the concurrence of quantum states. Compared with the calculation formula for concurrence, it does not need tomography of every element in the quantum state, saving a lot of physical resources. Moreover, the error of observing LU invariants is much smaller than that of tomography. For quantum states with a general structure, a more accurate prediction value of concurrence is obtained. The strong correlation between the concurrence and $I_{1}$, $I_{2}$, and $I_{14}$ and giving a lower bound on the concurrence by using $I_{1}$, $I_{2}$, and $I_{14}$ is a worthwhile thing to consider. In fact, $I_{2}$ has already given some theories on the lower bound on the concurrence degree, while the use of $I_{1}$ and $I_{14}$ to analyze the lower bound of concurrence presents a deeper research problem.

## Figures and Tables

**Table 1:** The table shows the local unitary invariants along with their corresponding variable names. Here, (a,b,c) stands for the triple scalar product a·(b×c), and $e_{ijk}$ is the Levi-Cevita symbol.

Variant	Formula	Variant	Formula
$I_{1,2,3}$	$\det\hat{\beta }$, $\mathrm{tr}({\hat{\beta }}^{T}\hat{\beta })$, $\mathrm{tr}{\left({\hat{\beta }}^{T}\hat{\beta }\right)}^{2}$	$I_{13}$	$s\hat{\beta }{\hat{\beta }}^{T}pp$
$I_{4,5,6}$	$s^{2}$, ${\left[s\hat{\beta }\right]}^{2}$, ${\left[s\hat{\beta }{\hat{\beta }}^{T}\right]}^{2}$	$I_{14}$	$e_{ijk}e_{lmn}s_{i}p_{l}\beta_{jm}\beta_{kn}$
$I_{7,8,9}$	$p^{2}$, ${\left[\hat{\beta }p\right]}^{2}$, ${\left({\hat{\beta }}^{T}\hat{\beta }p\right)}^{2}$	$I_{15}$	$\left(s,s\hat{\beta }{\hat{\beta }}^{T},\hat{\beta }p\right)$
$I_{10}$	$\left(s,s\hat{\beta }{\hat{\beta }}^{T},s{\left[\hat{\beta }{\hat{\beta }}^{T}\right]}^{2}\right)$	$I_{16}$	$\left(s\hat{\beta },p,{\hat{\beta }}^{T}\hat{\beta }p\right)$
$I_{11}$	$\left(p,{\hat{\beta }}^{T}\hat{\beta }p,{\left[{\hat{\beta }}^{T}\hat{\beta }\right]}^{2}p\right)$	$I_{17}$	$\left(s\hat{\beta },s\beta {\hat{\beta }}^{T}\hat{\beta },p\right)$
$I_{12}$	$s\hat{\beta }p$	$I_{18}$	$\left(s,\hat{\beta }p,\hat{\beta }{\hat{\beta }}^{T}\hat{\beta }p\right)$

**Table 2:** Evaluation results of 2-qubit pure state models.

	LR	Lasso	XGB	LGBM	BP-Net
RMSE	0.1570	0.0477	0.0002	0.0003	$1.57\times 10^{-5}$
$R^{2}$	0.9850	0.8779	0.9999	0.9999	0.9995

**Table 3:** Evaluation results of 2-qubit generated state models.

	LR	Lasso	XGB	LGBM	BP-Net
RMSE	0.0005	0.0018	0.0003	0.0003	0.0005
$R^{2}$	0.9452	0.8302	0.9665	0.9717	0.9488

**Table 4:** Table of tree model parameters.

Model	Hyperparameter	Value
XGBoost	Learning Rate	0.1
	Max Depth	7
	n_Estimator	500
LGBM	Learning Rate	0.1
	Max Depth	10
	Num Leaves	50
	n_Estimator	300

**Table 5:** Evaluation results of 2-qubit quantum state models.

States	Index	XGB	LGBM	BP-Net
G State	RMSE	0.0003	0.0001	0.0004
	R^2^	0.9664	0.9855	0.9535
P State	RMSE	$3.16\times 10^{-6}$	$6.67\times 10^{-6}$	$3.06\times 10^{-5}$
	R^2^	0.9999	0.9999	0.9992
W State	RMSE	$3.71\times 10^{-6}$	$7.2\times 10^{-6}$	$9.05\times 10^{-6}$
	R^2^	0.9999	0.9999	0.9999

## Data Availability

No data were used for the research described in this article.
